# Media exposure, perceived stereotype accessibility, and national image formation: experimental and short-term panel evidence from Chinese perceptions of Germany

**DOI:** 10.3389/fpsyg.2026.1858109

**Published:** 2026-09-04

**Authors:** Zihan Chen, Wenpei Sun

**Affiliations:** School of Marxism, China University of Geosciences (Wuhan), Wuhan, China

**Keywords:** competence and warmth, media exposure, media literacy, national stereotypes, perceived stereotype accessibility

## Abstract

**Background:**

In highly mediated environments, individuals often form impressions of foreign countries through indirect information rather than direct experience. Drawing on social cognition theory, this study examines how media exposure contributes to national stereotype formation through perceived stereotype accessibility and whether media literacy functions as a boundary condition.

**Methods:**

Three complementary studies were conducted among Chinese university students using Germany as the empirical case. Study 1 developed and preliminarily validated a context-specific measure of perceived stereotype accessibility and assessed the measurement properties of the core constructs. Study 2 employed an experimental design to test the causal effects of media exposure on perceived stereotype accessibility and stereotype content. Study 3 used a two-wave short-term panel design to examine the proposed relationships under temporal separation.

**Results:**

Across the studies, media exposure did not directly shape overall national attitudes. Instead, it increased the perceived accessibility of stereotype-related representations, which subsequently influenced national stereotype content. This indirect effect was primarily reflected in competence stereotypes rather than warmth stereotypes and was replicated across experimental and short-term panel contexts. Media literacy weakened the effect of media exposure on perceived stereotype accessibility, thereby reducing the downstream influence of media exposure on stereotype formation.

**Conclusion:**

The findings identify perceived stereotype accessibility as a key cognitive mechanism linking media exposure to national stereotype formation and demonstrate media literacy as an important boundary condition. The study contributes to research on media effects and national stereotyping by clarifying how mediated information becomes cognitively accessible and shapes stereotype content.

## 1 Introduction

In contemporary media environments, individuals increasingly form perceptions of foreign countries through indirect information rather than direct experience. This tendency is particularly pronounced among university students, many of whom have limited opportunities for sustained cross-cultural contact. Foreign countries are therefore often encountered through news coverage, audiovisual narratives, and social media content rather than firsthand interaction. These mediated perceptions may gradually develop into relatively stable national stereotypes that influence social judgments, emotional responses, and cross-cultural behavioral intentions (Dai et al., 2026; Fiske and Taylor, 2020).

Social psychological research has long emphasized that stereotypes do not simply mirror social reality. Instead, they function as cognitive simplification mechanisms under conditions of incomplete information and limited cognitive resources (Macrae and Bodenhausen, 2000). When direct contact is limited, individuals are more likely to rely on external informational cues to understand social groups. Media environments are especially influential because of their repetitive, structured, and contextualized representations (Efstratiou and De Cristofaro, 2022). By repeatedly associating social groups with particular traits, behaviors, or symbols, media representations can strengthen the cognitive associations between a group and specific attributes, making those associations more readily available in subsequent judgment (Roskos-Ewoldsen and Roskos-Ewoldsen, 2009).

From a social-cognitive perspective, media influence may operate not only through explicit persuasion or direct attitude change, but also through changes in cognitive accessibility (Arendt, 2023). Accessibility-based accounts suggest that information that is more easily activated and retrieved from memory is more likely to guide subsequent evaluation (Higgins, 1996; Hütter, 2022). Repeated exposure to social cues can therefore increase the accessibility of related stereotype representations and influence later judgments (Oğuz Taşbaş and Unkelbach, 2022). Accessibility-based processes have also been considered in research on national images, country stereotypes, and media representations. However, comparatively less research has integrated media exposure, perceived stereotype accessibility, stereotype content, and national attitudes within a single individual-level process model.

The stereotype content model provides a complementary framework for examining how accessible representations are translated into national evaluations. The model proposes that social groups are primarily evaluated along the dimensions of competence and warmth (Fiske, 2012). This two-dimensional structure has been validated across cultural contexts and shown to predict group attitudes, social distance, and willingness to cooperate (Cuddy et al., 2009). Although the model has been applied to a wide range of social categories, further examination is needed regarding how media exposure and stereotype accessibility jointly contribute to competence- and warmth-based judgments when the target is a nation as an abstract collective entity.

Communication studies and political psychology have produced extensive research on national image, country stereotypes, and media narratives. This literature has generated important insights into how countries are represented and how such representations relate to public opinion (Boscarino, 2022; Okechukwu, 2023; Tao and Peng, 2023). Building on this work, the present study focuses more specifically on the individual-level cognitive pathway through which media exposure is translated into stereotype content and broader national attitudes. It also addresses the methodological limitations of relying primarily on cross-sectional evidence by combining measurement development, experimental testing, and a two-wave short-term panel study.

Germany provides a theoretically informative context for examining this process. Its international image has undergone visible shifts in transnational public discourse. In the late 1990s and early 2000s, Germany was often described as the “sick man of Europe” because of weak economic growth and high unemployment (Dustmann et al., 2014). It was subsequently portrayed as a major economic power, while more recent public debate has again emphasized stagnation, crisis, and declining competitiveness (Bâlgăr, 2023). These changing representations illustrate how national images can be reconstructed through evolving media narratives rather than remaining fixed over time.

Chinese university students constitute an appropriate population for examining this process because their perceptions of foreign countries are often shaped more by mediated contact than by sustained firsthand experience. Prior research has shown that mediated contact plays an important role in shaping Chinese undergraduates’ perceptions of people and cultures beyond their immediate experience (Cao and Meng, 2020). Because many students have limited direct contact with Germany or German nationals, Germany is often encountered through news, audiovisual content, and online discussion. This combination of high media visibility and limited direct familiarity provides an appropriate setting for examining how media exposure relates to perceived stereotype accessibility, national stereotype content, and broader attitudes.

The present research makes three theoretical contributions. First, it moves beyond a direct-effects perspective by identifying perceived stereotype accessibility as a cognitive mechanism through which media exposure is translated into national stereotype content and subsequent attitudes. Second, it extends accessibility-based stereotyping research by examining nations as abstract collective targets. The findings further show that this process operates more consistently through competence than warmth, suggesting that the diagnostic value of stereotype dimensions varies across social targets and information environments. Third, the study conceptualizes media literacy as an early-stage cognitive filter that weakens the transformation of repeated media exposure into accessible stereotype representations rather than treating it merely as a direct predictor of attitudes.

## 2 Theoretical background and hypotheses

### 2.1 Media exposure and perceived stereotype accessibility

In cross-cultural cognitive contexts, individuals’ understanding of foreign countries is often grounded in limited and indirect sources of information. Social cognition theory suggests that when a social target is characterized by a lack of direct interaction experience, individuals are more inclined to rely on external informational cues to construct cognitive representations of that target (Fiske and Taylor, 2020). Compared with face-to-face contact, media information is marked by high repeatability, contextualization, and structural consistency, which makes it particularly influential in shaping social cognition. Through the continuous presentation of national behaviors, cultural symbols, and prototypical images, media do more than transmit information. They also subtly shape the cognitive pathways through which social targets are understood and evaluated (Flusberg et al., 2024).

From the perspective of cognitive processing, the primary impact of media on social cognition does not lie in directly changing attitudes, but rather in shaping cognitive accessibility (Sanborn, 2022). The frequency with which information is activated in memory and the ease with which it can be retrieved play a central role in determining which cues individuals rely on when making judgments (Higgins, 1996). When a social target and its associated traits are repeatedly activated, these representations become more readily available during subsequent cognitive processing and are therefore more likely to dominate judgment processes. Rather than systematically integrating all relevant information, individuals often rely on the most easily retrievable cues as the basis for evaluation (Anderson, 1990).

A substantial body of research across multiple domains of social cognition has provided empirical support for this mechanism. Rahmani Azad et al. (2023), for instance, demonstrated that stereotype-related information, once primed, can be automatically activated in subsequent judgment tasks, even in the absence of conscious awareness. Similarly, Bitektine et al. (2025) showed that repeated exposure to specific social cues increases the accessibility of related stereotypes, thereby amplifying their influence on social judgments. In the media context, sustained or repeated representations have also been shown to alter the relative salience of social category-related representations within the cognitive system (Roskos-Ewoldsen and Roskos-Ewoldsen, 2009). Taken together, these findings suggest that media influence social judgment not through explicit persuasion, but by reshaping the relative accessibility of competing cognitive representations.

This mechanism becomes particularly salient in national-level social cognition. Compared with concrete social groups such as ethnic or occupational categories, nations constitute more abstract social targets characterized by greater psychological distance. As a result, individuals often find it difficult to form systematic understandings of foreign countries through direct experience alone (Fiske, 2012). Under such conditions, media emerge as a primary source through which individuals acquire impressions of countries and their citizens. News reports, film and television portrayals, social media content, and everyday narratives provide repeated information about Germany and Germans, increasing the availability of Germany-related representations during subsequent judgments. When these cues are recurrently presented within the media environment, trait representations associated with the German group are more likely to be activated within the cognitive system, leading to higher levels of perceived stereotype accessibility.

Importantly, this process does not depend on whether media content carries explicit evaluative or persuasive intent. Even relatively neutral or descriptive social information can, through sufficient repetition, enhance the accessibility of related cognitive representations via cumulative effects (Verhoest et al., 2025). As perceived stereotype accessibility increases, stereotype-related impressions may be experienced as more readily available when individuals evaluate Germany. This subjective retrieval experience can increase the likelihood that readily available representations enter subsequent judgments. In the present research, perceived stereotype accessibility is examined at the level of self-reported retrieval experience. Behavioral indicators such as response latency, lexical-decision tasks, and sequential priming provide complementary approaches for examining automatic cognitive accessibility (Zhang et al., 2023).

Perceived stereotype accessibility refers to individuals’ subjective experience of how readily stereotype-related impressions come to mind when they think about a social group. It captures perceived retrieval ease, immediacy, and judgmental fluency during group evaluation. Stereotype content represents the substantive characteristics attributed to the target, with competence and warmth serving as the two focal dimensions in the present study. Stereotype strength concerns properties of these evaluations, such as their certainty, extremity, or stability. These constructs therefore describe different aspects of stereotype-related cognition: perceived stereotype accessibility concerns the experienced ease of bringing relevant impressions to mind, stereotype content concerns what characteristics are attributed to the group, and stereotype strength concerns how firmly those evaluations are held.

Accordingly, the following hypothesis is proposed:

*H1*: Greater exposure to Germany-related media content is associated with higher levels of perceived stereotype accessibility regarding the German group.

### 2.2 Perceived stereotype accessibility and national stereotype content

If media exposure influences individuals’ cognitive starting points by increasing perceived stereotype accessibility, a further question concerns how such accessibility translates into concrete judgments of stereotype content. Stereotype accessibility does not merely determine whether a particular social representation is activated. It also shapes the evaluative dimensions individuals rely on during judgment and the relative weight assigned to those dimensions (Higgins, 1996). When trait representations associated with a social target are more easily retrievable, individuals are more likely to draw on these activated cues in group evaluation, leading to more coherent and consistent judgment patterns.

The stereotype content model provides a central theoretical framework for understanding this process. According to the model, evaluations of social groups are primarily structured along two fundamental dimensions: competence and warmth (Fiske et al., 2007). Competence reflects perceptions of a group’s ability, efficiency, and control over resources, whereas warmth captures perceived intentions, such as friendliness and trustworthiness. These dimensions are regarded as the most basic and universally applicable criteria in social perception, and they reliably predict emotional reactions, social distance, and willingness to cooperate with social groups (Cuddy et al., 2008).

From a cognitive-processing perspective, stereotype content is not generated spontaneously at the moment of judgment. Rather, it relies on pre-existing trait representations stored within the cognitive system. When trait representations associated with a social target become more accessible, individuals can retrieve this information more rapidly during evaluation, resulting in more pronounced judgments along the corresponding dimensions (Higgins et al., 2022). Increased accessibility makes the retrieved representations more likely to be used as inputs when individuals form competence- and warmth-related judgments (Ye et al., 2026).

This mechanism is particularly salient in national-level social cognition. Nations constitute highly abstract social targets, and individuals often lack sufficient direct experience to form comprehensive understandings of them. Consequently, judgments tend to rely more heavily on readily accessible trait cues (Rueschemeyer and Skocpol, 2017). When trait representations related to a national group are easily activated, evaluations along the competence and warmth dimensions are more likely to be anchored in these activated cues. Even in the absence of systematic information, individuals can quickly form judgments about countries or their citizens on these two dimensions and use such evaluations as the basis for subsequent attitudes and behavioral intentions (Fiske et al., 2007).

Evidence from cross-cultural research further indicates that competence and warmth exhibit a high degree of structural stability across cultural contexts (Grigoryev, 2022). This suggests that when stereotype accessibility increases, its influence on stereotype content is not random. Instead, accessibility effects are more likely to manifest through these two core dimensions. In this sense, accessibility does not determine the evaluative direction per se, but rather determines which dimensions and traits are most readily used in evaluation.

Integrating these theoretical considerations, the present study argues that higher levels of stereotype accessibility regarding the German group increase the likelihood that individuals will rely on activated trait representations when forming evaluations. As a result, judgments along the competence and warmth dimensions become more salient and pronounced.

Although competence and warmth are both fundamental dimensions of social perception, they may not be equally responsive to mediated information. Media representations of nations frequently emphasize economic performance, technological capability, institutional efficiency, and international influence, all of which provide relatively diagnostic cues for competence judgments. Warmth judgments, by contrast, depend more strongly on perceived intentions, emotional closeness, and direct interpersonal contact. Accordingly, media-based stereotype accessibility may be more readily translated into competence evaluations than into warmth evaluations when the target is a nation.

Accordingly, the following hypotheses are proposed:

*H2a*: Higher levels of perceived stereotype accessibility are associated with more positive warmth evaluations of the German group.

*H2b*: Higher levels of perceived stereotype accessibility are associated with more positive warmth evaluations of the German group.

### 2.3 Stereotype content and social attitudes toward a nation

The stereotype content model not only explains how individuals form cognitive evaluations of social groups, but has also been widely applied to predict the emotional responses and behavioral tendencies that follow from these evaluations. A substantial body of research demonstrates that competence and warmth are not merely descriptive judgments. Rather, they constitute core psychological foundations underlying group attitudes and behavioral intentions (Fiske et al., 2007; Cuddy et al., 2008; Eisenbruch and Krasnow, 2022). Once individuals develop relatively clear stereotypes along these dimensions, such evaluations tend to shape their overall attitudes toward the group and their willingness to engage with its members.

Competence stereotypes are typically linked to judgments of a group’s social status, efficiency, and reliability, and have been shown to predict respect, trust, and expectations of effective cooperation (Fiske et al., 2007). In the context of national-level social cognition, perceptions of a country’s competence often inform judgments about national strength, institutional effectiveness, and cooperative value, thereby shaping more instrumental and rational attitudes toward that country (Kervyn et al., 2015).

In contrast, warmth stereotypes are more closely tied to affective social judgments. Warmth is the most direct predictor of liking, perceived closeness, and social distance, and its influence often exceeds that of competence, particularly in contexts involving interpersonal intentions and emotional responses (Cuddy et al., 2008; Fiske, 2018). When a national group is perceived as friendly, trustworthy, and well-intentioned, individuals are more likely to develop positive affective attitudes and display stronger willingness to engage and interact.

When nations serve as social targets, competence and warmth similarly function as foundational dimensions shaping overall attitudes. Even under conditions of limited information, individuals tend to rapidly evaluate countries or their citizens along these two dimensions and rely on such evaluations in subsequent judgments and decisions (Fiske et al., 2007; Kunst et al., 2025). Accordingly, when individuals hold salient competence and warmth stereotypes about the German group, these cognitive evaluations are likely to influence their overall attitudes toward Germany.

Based on this theoretical reasoning, the present study posits that stereotype content carries significant psychological consequences in national cognition. Specifically, more positive competence and warmth stereotypes are expected to be associated with more favorable overall attitudes toward Germany. Thus, the following hypotheses are proposed:

*H3a*: More positive competence stereotypes regarding the German group are associated with more positive overall attitudes toward Germany.

*H3b*: More positive warmth stereotypes regarding the German group are associated with more positive overall attitudes toward Germany.

### 2.4 The mediating role of perceived stereotype accessibility

Integrating the preceding theoretical arguments, the present study contends that media do not shape national stereotype content in a direct manner. Rather, media exert their influence indirectly by altering the accessibility of relevant cognitive representations, which in turn guides the formation of stereotype content. Accessibility functions as a key psychological mechanism linking external information environments to internal judgment processes, determining which representations are most likely to enter evaluative consideration (Higgins et al., 2022; Unkelbach et al., 2023). This mechanism becomes particularly consequential in cross-cultural contexts where media constitute a dominant source of social information.

Prior research suggests that media exposure can indirectly influence group judgments and attitudinal evaluations by increasing the accessibility of specific social representations (Roskos-Ewoldsen and Roskos-Ewoldsen, 2009). Work grounded in the stereotype content model further indicates that judgments of competence and warmth are often anchored in activated cognitive cues rather than derived from systematic integration of all available information (Fiske et al., 2007). Together, these perspectives imply that stereotype accessibility is likely to serve as a psychological bridge between media exposure and stereotype content.

Bringing these two theoretical strands together, it can be inferred that in national-level social cognition, media exposure first elevates the accessibility of trait representations associated with the German group, thereby shifting the cognitive starting point individuals rely on during judgment. These activated representations are then translated into stereotype content judgments along the competence and warmth dimensions. In this sense, stereotype accessibility constitutes a central psychological pathway through which media environments shape national stereotype content.

Based on this integrative framework, the present study proposes the following mediation hypotheses:

*H4a*: Perceived stereotype accessibility mediates the relationship between exposure to Germany-related media content and competence stereotypes regarding the German group.

*H4b*: Perceived stereotype accessibility mediates the relationship between exposure to Germany-related media content and warmth stereotypes regarding the German group.

### 2.5 The moderating role of media literacy

Although the preceding sections establish a psychological pathway through which media exposure enhances stereotype accessibility and subsequently shapes national stereotype content, this process rests on an implicit assumption: that individuals incorporate media-presented social information into cognitive processing in a relatively direct manner. Social cognition research, however, has repeatedly demonstrated that external information does not exert uniform effects across individuals. Identical stimuli can elicit markedly different psychological responses depending on characteristics of the cognitive perceiver (Biró et al., 2022). Ignoring such individual differences risks oversimplifying models of media influence.

In contemporary media-saturated environments, individuals are not passive recipients of information. Instead, they engage with media content with varying degrees of reflexivity and critical awareness (Petrova and Tapsoba, 2025). Some individuals are attuned to the constructed nature of media representations, recognize discrepancies between mediated portrayals and social reality, and maintain psychological distance from media cues during cognitive processing. Others, by contrast, are more inclined to treat repeatedly encountered media representations as direct reflections of reality. These differences play a decisive role in determining whether media information is transformed into highly accessible stereotype representations.

Media literacy captures this distinction at a psychological level. Unlike mere frequency of media use, media literacy emphasizes individuals’ ability to understand media sources, presentation strategies, and potential biases, as well as their capacity for critical evaluation (Potter, 2004; Hobbs, 2024). Individuals with higher media literacy are more likely to engage in metacognitive processing of media content, treating it as information that requires interpretation and scrutiny rather than as unproblematic social fact. Such processing reduces the likelihood that repeated media exposure will produce automatic cognitive associations, thereby attenuating increases in stereotype accessibility.

From an information-processing perspective, this dynamic can be understood as the regulation of automatic social judgment. Although stereotype activation often occurs automatically, its influence on judgment is not beyond cognitive control (Bodenhausen and Macrae, 2013; Devine and Sharp, 2009). Individuals with stronger reflective capacities are better able to revise or inhibit activated stereotypes, reducing the weight these representations carry in evaluation. Media-literate individuals operate through this mechanism, weakening the accessibility advantage conferred by media exposure and preventing stereotype representations from occupying a dominant position within the cognitive system.

By contrast, individuals with lower levels of media literacy are more likely to process media information heuristically (Traberg et al., 2024). Recurrently presented social representations can be rapidly encoded and stored as judgment cues, which are then readily retrieved in subsequent cognition involving national targets. This mode of processing not only increases stereotype accessibility but also amplifies its influence on social judgment. Such differences are especially pronounced when nations serve as highly abstract social targets, as the absence of direct experience increases reliance on mediated cues.

Taken together, these considerations suggest that media literacy does not merely shape whether individuals are exposed to media, but more fundamentally moderates how media exposure is translated into cognitive accessibility. The effect of media exposure on stereotype accessibility is therefore neither uniform nor universal, but systematically contingent on individuals’ levels of media literacy. Accordingly, the following moderation hypothesis is proposed:

*H5*: Media literacy moderates the relationship between exposure to Germany-related media content and perceived stereotype accessibility, such that the positive effect of media exposure on perceived stereotype accessibility is weaker among individuals with higher media literacy than among those with lower media literacy.

**Figure 1:**
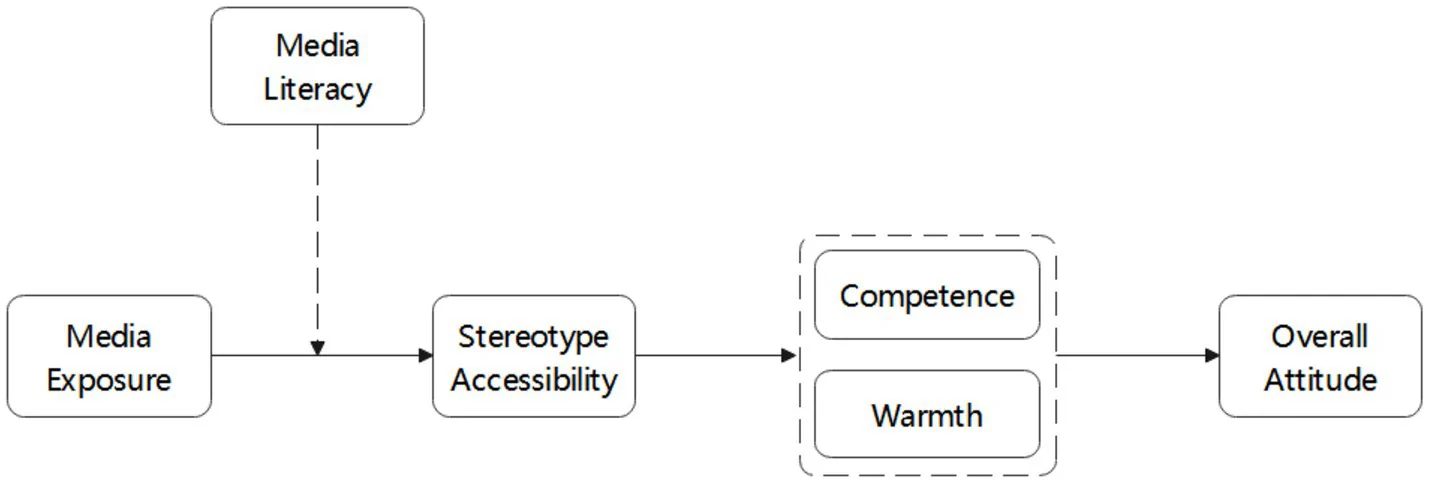
Research model.

The research model is shown in Figure 1:

## 3 Methods

The present research employed a multi-study design to systematically examine how media shape national stereotype content through the mechanism of perceived stereotype accessibility and how these cognitive processes further influence social attitudes toward a nation. The research program was structured across three complementary studies, addressing measurement construction, causal inference, and validation within natural media environments. Study 1 focused on establishing the measurement structure and discriminant validity of the core constructs. Study 2 adopted an experimental approach to test the causal effects of media exposure intensity and the underlying psychological mechanisms. Study 3 employed a two-wave short-term panel design to examine whether the proposed relationships could be replicated under temporal separation in a naturally occurring media environment. All studies were conducted in accordance with academic ethical standards and received ethical approval prior to data collection.

National stereotype content was assessed using competence and warmth measures adapted from the stereotype content model (Fiske et al., 2007; Cuddy et al., 2008). Media literacy was measured using items adapted from Potter’s (2004) conceptualization and subsequent empirical research (Jeong et al., 2012). Because no established self-report scale directly captured perceived stereotype accessibility in the context of national stereotypes, this measure was systematically developed and preliminarily validated in Study 1. The measures of Germany-related media exposure and overall attitude toward Germany were constructed for the present context based on relevant conceptualizations in prior research and were subsequently evaluated together with the other measures in Study 1.

### 3.1 Study 1: method

Study 1 was conducted as a measurement development and preliminary validation study to establish the measures used in the subsequent experimental and short-term panel studies. The competence, warmth, and media literacy measures were adapted from previously published research and contextualized for perceptions of Germany. The media exposure and overall-attitude items were constructed for the present national-image context based on relevant conceptualizations in prior research. Because no existing self-report scale directly captured the perceived ease with which national stereotype representations are activated and retrieved, a context-specific measure of perceived stereotype accessibility was systematically developed.

The development of the perceived stereotype accessibility scale followed a multistage procedure. First, based on a review of research on cognitive accessibility, stereotype activation, memory retrieval, and judgment fluency, an initial pool of 14 items was generated. The items were designed to capture participants’ perceived ease and speed of bringing stereotype-related impressions to mind when thinking about Germans.

Second, four experts with research experience in social psychology, media studies, and psychometric measurement independently evaluated the initial items in terms of conceptual relevance, clarity, and redundancy. Based on their evaluations, three items were removed because they primarily reflected general knowledge or familiarity with Germany rather than retrieval accessibility, and two pairs of items with highly similar wording were combined. This process resulted in nine revised items.

Third, a cognitive pretest was conducted with 18 university students who were not included in the formal study. Participants completed the items and were subsequently asked to explain how they understood each statement and to identify any wording that was ambiguous, repetitive, or difficult to answer. One item was removed because participants interpreted it inconsistently as reflecting attitude certainty rather than accessibility, and several items were slightly reworded to improve clarity. The resulting eight-item pool was then administered in the formal quantitative pretest. Exploratory and confirmatory factor analyses were subsequently used to identify the final four-item measure.

Participants were undergraduate and graduate students enrolled at Chinese universities who voluntarily participated through an WJX.COM. Before completing the questionnaire, all participants read and confirmed an informed consent statement. Data were collected anonymously. Attention-check items were included, and responses with abnormally short completion times or evident response-pattern problems were excluded.

The quantitative pretest included measures of media exposure, perceived stereotype accessibility, competence, warmth, overall attitudes toward Germany, and media literacy. Perceived stereotype accessibility captured the subjective ease and speed with which stereotype-related representations were activated and retrieved when participants thought about Germans. Competence and warmth were measured using scales adapted from the stereotype content model, whereas overall attitudes toward Germany were included to assess discriminant validity. All items were measured using seven-point Likert scales.

The valid sample was randomly divided into two subsamples. Exploratory factor analysis was conducted using the first subsample to identify the factor structure and remove items with low loadings, substantial cross-loadings, or conceptual overlap. Confirmatory factor analysis was then conducted using the second subsample to evaluate model fit, factor loadings, internal consistency, composite reliability, and average variance extracted. Discriminant validity was assessed using the Fornell–Larcker criterion, HTMT ratios, and comparisons with alternative measurement models. The final items retained through this process were subsequently used in Study 2 and 3.

### 3.2 Study 2: method

Study 2 employed a between-subjects randomized experiment to test the causal effect of Germany-related media exposure on perceived stereotype accessibility and downstream stereotype content. Participants were randomly assigned to one of two conditions that differed in the intensity of exposure to Germany-related media information: a high-exposure condition and a low-exposure condition. In the high-exposure condition, participants read several short media texts concerning Germany and Germans across domains such as the economy, technology, and social life. In the low-exposure condition, participants were exposed to only limited Germany-related information, while the remaining reading materials were unrelated filler texts. All materials were matched in length, linguistic style, and information density. This design was intended to manipulate the amount of Germany-related exposure while minimizing evaluative framing differences across conditions.

After the reading task, participants completed manipulation-check items assessing perceived exposure to Germany-related information, followed by measures of perceived stereotype accessibility, competence stereotypes, warmth stereotypes, and overall attitudes toward Germany. Media literacy was measured prior to the experimental task and was modeled as an individual-level moderator of the relationship between media exposure and perceived stereotype accessibility.

Data analysis first examined whether the manipulation successfully created perceived differences in Germany-related media exposure across conditions. The hypothesized relationships were then tested using structural modeling and bootstrapping procedures to estimate direct, indirect, and moderated effects.

### 3.3 Study 3: method

Study 3 employed a two-wave short-term panel design to examine whether earlier Germany-related media exposure predicted later perceived stereotype accessibility and national evaluations under temporal separation. Participants were undergraduate students from Chinese universities. Data were collected at two time points separated by an interval of approximately 2 weeks. To match responses across waves while preserving anonymity, participants generated self-coded identifiers.

At Time 1, participants reported their levels of Germany-related media exposure and media literacy, together with baseline evaluations of Germany, including competence stereotypes, warmth stereotypes, and overall attitudes. At Time 2, perceived stereotype accessibility, competence stereotypes, warmth stereotypes, and overall attitudes toward Germany were assessed. Measurement items were consistent with those used in the previous studies to ensure comparability across the research program.

Data analysis relied on a time-lagged structural model. Time 1 media exposure was used to predict Time 2 perceived stereotype accessibility, which in turn was modeled as a predictor of Time 2 stereotype content and overall attitudes. Baseline competence stereotypes, warmth stereotypes, and overall attitudes measured at Time 1 were included as controls to reduce the influence of prior evaluations.

## 4 Results

### 4.1 Results of study 1

#### 4.1.1 Item screening and exploratory factor analysis

Following the literature review and expert evaluation, eight candidate items were retained for the quantitative assessment of perceived stereotype accessibility. A total of 320 valid responses were collected from undergraduate and graduate students through WJX.COM, a widely used online survey platform in China. The platform recorded completion time and restricted repeated submissions from the same device or IP address. The full sample was then randomly divided into two equal subsamples. The first subsample (*n* = 160) was used for exploratory factor analysis, and the second subsample (*n* = 160) was used for confirmatory factor analysis.

Exploratory factor analysis was conducted using principal axis factoring. The Kaiser–Meyer–Olkin value was 0.846, and Bartlett’s test of sphericity was statistically significant, χ^2^(28) = 623.41, *p* < 0.001, indicating that the data were suitable for factor analysis.

The initial analysis supported a dominant single-factor structure. Four items were subsequently removed because of low factor loadings, limited communalities, or conceptual overlap with familiarity, knowledge, evaluative certainty, and attitude stability. The remaining four items loaded clearly on one factor, with factor loadings ranging from 0.75 to 0.84. The final factor accounted for 63.68% of the variance (see Table 1).

**Table 1:** Exploratory factor analysis of perceived stereotype accessibility.

Item	Item description	Factor loading	Communality	Decision
SA1	When I think of Germans, certain typical characteristics immediately come to mind.	0.82	0.67	Retained
SA2	My impression of Germans is clear and well defined.	0.78	0.61	Retained
SA3	It is not difficult for me to describe what a typical German is like.	0.84	0.71	Retained
SA4	When I need to evaluate Germans, I can form a judgment very quickly.	0.75	0.56	Retained
SA5	I am familiar with common descriptions of Germans.	0.46	0.21	Removed
SA6	I have a strong overall impression of Germans.	0.49	0.24	Removed
SA7	I know a great deal about German society.	0.42	0.18	Removed
SA8	My views of Germans remain stable over time.	0.44	0.19	Removed

Extraction method: principal axis factoring. KMO = 0.846; Bartlett’s test: χ^2^(28) = 623.41, *p* < 0.001.

The deleted items were not retained because they primarily reflected familiarity, knowledge, evaluative strength, or temporal stability rather than the subjective ease and speed with which stereotype-related representations were activated and retrieved.

#### 4.1.2 Confirmatory factor analysis

Confirmatory factor analysis was conducted using the second subsample. The proposed six-factor measurement model included media exposure, perceived stereotype accessibility, competence, warmth, overall attitude toward Germany, and media literacy.

The six-factor model demonstrated satisfactory fit to the data, χ^2^(237) = 366.42, χ^2^/df = 1.55, CFI = 0.953, TLI = 0.945, RMSEA = 0.059, 90% CI [0.047, 0.070], and SRMR = 0.052. All standardized factor loadings were statistically significant and exceeded 0.60.

To further assess construct distinctiveness, the proposed six-factor model was compared with several alternative models. In particular, because perceived stereotype accessibility was moderately correlated with competence, an alternative five-factor model was estimated in which these two constructs were combined. The proposed six-factor model demonstrated significantly better fit than the alternative models, with all chi-square difference tests reaching statistical significance (all *p* < 0.001). These results further support the conceptual and empirical distinction between perceived stereotype accessibility, stereotype content, and overall national attitudes (see Table 2).

**Table 2:** Comparison of alternative measurement models.

Model	χ^2^	df	χ^2^/df	CFI	TLI	RMSEA	SRMR	Δχ^2^	Δdf	*p*
Six-factor model	366.42	237	1.55	0.953	0.945	0.059	0.052	—	—	—
Five-factor model: SA and CO combined	527.64	242	2.18	0.896	0.882	0.086	0.079	161.22	5	<0.001
Five-factor model: CO and WA combined	498.31	242	2.06	0.907	0.894	0.081	0.074	131.89	5	<0.001
Five-factor model: SA and AT combined	552.48	242	2.28	0.888	0.874	0.089	0.082	186.06	5	<0.001
Single-factor model	1120.65	252	4.45	0.681	0.648	0.147	0.139	754.23	15	<0.001

SA, perceived stereotype accessibility; CO, competence; WA, warmth; AT, overall attitude toward Germany. Δχ^2^ and Δdf represent chi-square difference tests relative to the six-factor model.

These results supported the distinction between perceived stereotype accessibility, stereotype content, and overall national attitudes.

#### 4.1.3 Reliability and convergent validity

The reliability and convergent validity results are presented in Table 3. Cronbach’s α values ranged from 0.80 to 0.86, and composite reliability values ranged from 0.83 to 0.87, exceeding the recommended threshold of 0.70.

**Table 3:** Reliability and convergent validity.

Construct	Items	Standardized loading range	Cronbach’s α	CR	AVE
Media exposure	4	0.66–0.81	0.80	0.83	0.55
Perceived stereotype accessibility	4	0.70–0.82	0.82	0.85	0.58
Competence	4	0.73–0.84	0.86	0.87	0.62
Warmth	4	0.71–0.82	0.84	0.85	0.59
Overall attitude toward Germany	3	0.76–0.86	0.85	0.85	0.66
Media literacy	5	0.67–0.80	0.84	0.86	0.56

CR, composite reliability; AVE, average variance extracted.

All average variance extracted values exceeded 0.50. The standardized factor loadings ranged from 0.66 to 0.86, providing evidence of satisfactory convergent validity.

#### 4.1.4 Discriminant validity

Discriminant validity was first examined using the Fornell–Larcker criterion. As shown in Table 4, the square root of the AVE for each construct exceeded its correlations with all other constructs. In addition, all HTMT values were below the recommended threshold of 0.85, providing further support for discriminant validity.

**Table 4:** Fornell–Larcker criterion for Study 1.

Construct	ME	SA	CO	WA	AT	ML
ME	0.74	0.235	0.155	0.125	0.150	0.186
SA	0.235	0.76	0.483	0.353	0.387	−0.035
CO	0.155	0.483	0.79	0.354	0.479	−0.020
WA	0.125	0.353	0.354	0.77	0.462	−0.048
AT	0.150	0.387	0.479	0.462	0.81	−0.003
ML	0.186	−0.035	−0.020	−0.048	−0.003	0.75

ME, media exposure; SA, perceived stereotype accessibility; CO, competence; WA, warmth; AT, overall attitude toward Germany; ML, media literacy.

Although perceived stereotype accessibility was moderately correlated with competence (r = 0.483), the two constructs shared only 23.3% of their variance. Moreover, the square roots of their AVE values exceeded their correlation, the corresponding HTMT value was below 0.85, and the model combining the two constructs demonstrated substantially poorer fit than the proposed six-factor model. These results support their conceptual and empirical distinctiveness.

#### 4.1.5 Finalization of the measures

The results of Study 1 supported the reliability and validity of the measurement instruments. In particular, the four retained perceived stereotype accessibility items demonstrated satisfactory factor loadings, internal consistency, convergent validity, and discriminant validity.

The final four-item perceived stereotype accessibility scale, together with the adapted competence, warmth, and media literacy measures and the context-specific measures of media exposure and overall attitude toward Germany, was subsequently used in Studies 2 and 3 without further modification.

### 4.2 Results of study 2

#### 4.2.1 Objective

Building on Study 1, which established the reliability, structural validity, and basic correlational patterns of the key constructs, Study 2 employed an experimental design to examine the proposed theoretical relationships at the causal level. Specifically, Study 2 aimed to test whether media exposure influences Chinese university students’ national stereotype content regarding Germany through perceived stereotype accessibility, and whether this process further shapes their overall attitudes toward Germany. In addition, this study examined the moderating role of media literacy as an individual-level boundary condition in the formation of national stereotypes.

By introducing experimental manipulation, Study 2 sought to overcome the inherent limitations of cross-sectional survey designs in causal inference and to provide more rigorous empirical support for the proposed theoretical model.

#### 4.2.2 Method

Study 2 adopted a single-factor experimental design in which the intensity of Germany-related media exposure was manipulated to examine its impact on stereotype formation. Participants were undergraduate and graduate students from Chinese universities who voluntarily took part in the study through WJX.COM, a widely used online survey and experimental research platform in China. Participants were randomly assigned to the experimental conditions using the platform’s random assignment function. WJX.COM also enabled controlled presentation of the experimental materials, response-time recording, attention checks, and restrictions on repeated submissions from the same device or IP address.

An *a priori* power analysis conducted using G*Power indicated that a minimum sample of 200 participants was required for a two-group between-subjects design, assuming a two-tailed test, an effect size of d = 0.40, α = 0.05, and power of 0.80. Allowing for approximately 15% attrition or invalid responses, the target sample size was set at 235 participants. Accordingly, a total of 236 participants were initially recruited. After excluding incomplete responses, duplicate submissions, responses with abnormally short completion times, and participants who failed the attention-check items, the final sample consisted of 212 valid cases. The sample included 47.6% male and 52.4% female participants, with a mean age of 20.84 years (SD = 1.92). Participants came from diverse academic backgrounds, including social sciences and humanities (31.1%), business-related majors (26.9%), engineering and natural sciences (29.7%), and other fields, providing a reasonably heterogeneous representation of the Chinese university student population.

All participants were informed of the study purpose and procedure prior to participation and confirmed that their participation was entirely voluntary. No personally identifiable information was collected, and all data were recorded and analyzed anonymously in accordance with academic ethical standards. Participants were randomly assigned to experimental conditions to minimize systematic background differences across groups.

The experimental stimuli consisted of six short simulated news articles, each containing approximately 130–150 words, with a total length of approximately 840 words in both conditions. In the high media exposure condition, all six articles concerned Germany or Germans and covered topics including economic development, manufacturing and technology, education and vocational training, social welfare and demographic change, environmental and energy policy, and everyday social and cultural life. In the low media exposure condition, participants read one Germany-related article and five filler articles unrelated to Germany. The filler articles addressed neutral topics such as general science, transportation, food production, and everyday technology.

The materials were developed by the researchers through the synthesis and paraphrasing of publicly available factual reports and news content rather than being reproduced verbatim from a single source. Source names, logos, and other identifying information were removed to minimize the potential influence of source credibility. All texts were written in a neutral and descriptive style and avoided explicitly favorable or unfavorable evaluations of Germany. Across conditions, the materials were matched as closely as possible in total word count, sentence length, linguistic complexity, presentation format, and approximate reading time. The primary difference between the two conditions was therefore the amount of Germany-related information presented. The manipulation therefore focused specifically on the amount of Germany-related information presented, while stereotype consistency was not experimentally varied.

A sample paragraph from the Germany-related materials was as follows: “Germany’s economy includes a large manufacturing sector alongside service industries. Automobile production, mechanical engineering, chemicals, and business services contribute to employment and international trade. Public discussion has also addressed energy costs, demographic change, and the adjustment of industrial production. These developments have created both opportunities and pressures for German firms and public institutions.” Representative sample paragraphs are provided in Supplementary Appendix B.

After completing the reading task, participants completed questionnaires measuring perceived stereotype accessibility (SA), competence stereotypes (CO), warmth stereotypes (WA), and overall attitudes toward Germany (AT). Media literacy (ML) was measured prior to the experimental task as an individual difference variable to examine its moderating role in the relationship between media exposure and perceived stereotype accessibility. All variables were measured using seven-point Likert scales, and the items were identical to those used in Study 1.

#### 4.2.3 Results

Before testing the structural model, manipulation checks were conducted to assess the effectiveness of the experimental manipulation. The results showed that participants in the high Germany-related media exposure condition reported significantly higher perceived exposure to Germany-related information than those in the low exposure condition, indicating that the manipulation functioned as intended. Participants in the high-exposure condition reported significantly greater Germany-related media exposure (M = 4.768) than those in the low-exposure condition (M = 3.411), t(210) = 6.671, *p* < 0.001, Cohen’s d = 0.924.

After confirming satisfactory measurement model fit, the structural model was examined. Overall model fit indices indicated a good fit between the theoretical model and the observed data. All major fit indices met commonly accepted standards in psychological research (χ^2^/df < 3, CFI and TLI > 0.90, RMSEA and SRMR < 0.08), suggesting that the proposed structural model adequately captured the relationships among the study variables.

As shown in Table 5, structural path analyses revealed a significant positive effect of media exposure on perceived stereotype accessibility, indicating that increased exposure to Germany-related media information increased participants’ perceived ease of retrieving stereotype-related representations associated with Germany. Further analyses showed that perceived stereotype accessibility had a significant positive effect on competence stereotypes. In contrast, its effect on warmth stereotypes was weaker and did not reach statistical significance. This pattern suggests that perceived stereotype accessibility does not exert uniform effects across the two dimensions of national stereotype content, with a more pronounced influence on competence-related judgments.

**Table 5:** SEM of study 2.

Path	β	SE	z	*p*	f^2^	Hypothesis
ME → PSA	0.412	0.069	5.971	<0.001	0.119	H1 supported
SA → CO	0.523	0.064	8.172	<0.001	0.449	H2a supported
SA → WA	0.118	0.078	1.513	0.130	0.036	H2b not supported
CO → AT	0.458	0.071	6.451	<0.001	0.091	H3a supported
WA → AT	0.287	0.059	4.864	<0.001	0.039	H3b supported
PSA → AT	0.062	0.063	0.984	0.325	0.033	—
ME → AT	0.041	0.051	0.804	0.421	0.004	—
ME × ML → SA	−0.176	0.068	−2.588	0.010	0.033	H5 supported

Model fit indices: χ^2^/df = 2.140; CFI = 0.930; TLI = 0.920; RMSEA = 0.061; SRMR = 0.049.

Regarding the relationship between stereotype content and overall attitudes, both competence and warmth stereotypes had significant positive effects on overall attitudes toward Germany. In contrast, neither the direct effect of perceived stereotype accessibility nor the direct effect of media exposure on overall attitudes was statistically significant. Together, these findings suggest that media exposure primarily influences national attitudes through stereotype-related cognitive processes rather than through a direct attitudinal pathway.

The explanatory and predictive power of the model was also examined. The model explained 13.1% of the variance in perceived stereotype accessibility, 31.0% of the variance in competence stereotypes, 3.5% of the variance in warmth stereotypes, and 27.0% of the variance in overall attitudes toward Germany. All Stone–Geisser Q^2^ values were above zero, indicating predictive relevance for the endogenous constructs, although predictive relevance was relatively limited for warmth. The f^2^ results further showed that perceived stereotype accessibility made a substantial incremental contribution to competence stereotypes, whereas its contribution to warmth stereotypes was considerably smaller. The direct contribution of media exposure to overall attitudes was negligible (see Table 6).

**Table 6:** Explanatory and predictive power of the study 2 model.

Endogenous construct	R^2^	Q^2^	Interpretation
Perceived stereotype accessibility	0.131	0.109	Modest explanatory and predictive relevance
Competence	0.310	0.301	Moderate explanatory and predictive relevance
Warmth	0.035	0.017	Limited explanatory and predictive relevance
Overall attitude toward Germany	0.270	0.235	Moderate explanatory and predictive relevance

Building on the structural path analysis, we further examined the mediating role of perceived stereotype accessibility in the relationship between media exposure and the formation of national cognition and attitudes. As shown Table 7, bootstrapping analyses with 5,000 resamples revealed a significant indirect effect of media exposure on competence stereotypes through perceived stereotype accessibility (indirect effect = 0.215, SE = 0.041, 95% CI [0.142, 0.302]). This result indicates that, within the experimental context, higher levels of media exposure strengthened competence-related judgments of the German group by increasing the cognitive accessibility of relevant stereotypes. In contrast, the indirect effect of media exposure on warmth stereotypes via perceived stereotype accessibility did not reach statistical significance (indirect effect = 0.049, SE = 0.033, 95% CI [−0.011, 0.118]), as the confidence interval included zero.

**Table 7:** Indirect effects and moderating mediating effects of Study 2 (Bootstrap = 5,000).

Indirect path		Indirect effect	SE	95% CI	Significant
ME → SA → CO		0.215	0.041	[0.142, 0.302]	H4a Significant
ME → SA → WA		0.049	0.033	[−0.011, 0.118]	H4b Not Significant
ME → SA → CO → AT		0.098	0.024	[0.053, 0.147]	-
ME → SA → WA → AT		0.018	0.014	[−0.004, 0.046]	-
ME → SA → AT		0.009	0.011	[−0.012, 0.031]	-
Indirect path	Media literacy level	Conditional indirect effect	SE	95% CI	Significant
ME → SA → CO	Low ML (−1 SD)	0.231	0.046	[0.148, 0.329]	Significant
	High ML (+1 SD)	0.141	0.039	[0.074, 0.228]	Significant
ME → SA → WA	Low ML (−1 SD)	0.072	0.038	[−0.001, 0.151]	Not Significant
	High ML (+1 SD)	0.031	0.029	[−0.024, 0.098]	Not Significant
ME → SA → CO → AT	Low ML (−1 SD)	0.108	0.028	[0.056, 0.168]	Significant
	High ML (+1 SD)	0.065	0.022	[0.026, 0.114]	Significant
ME → SA → WA → AT	Low ML (−1 SD)	0.021	0.017	[−0.006, 0.057]	Not Significant
	High ML (+1 SD)	0.009	0.012	[−0.013, 0.034]	Not Significant

Indirect effects were evaluated using 5,000 bootstrap resamples; effects were considered statistically significant when the 95% confidence interval excluded zero.

We further conducted a serial mediation analysis with overall national attitude as the outcome variable. The results showed a significant indirect effect of media exposure on overall attitudes through perceived stereotype accessibility and competence stereotypes (ME → SA → CO → AT: indirect effect = 0.098, SE = 0.024, 95% CI [0.053, 0.147]). By contrast, the corresponding serial mediation pathway through perceived stereotype accessibility and warmth stereotypes was not statistically significant (ME → SA → WA → AT: indirect effect = 0.018, SE = 0.014, 95% CI [−0.004, 0.046]). In addition, the single-mediator pathway from media exposure to overall attitude through perceived stereotype accessibility alone was also non-significant (ME → SA → AT: indirect effect = 0.009, SE = 0.011, 95% CI [−0.012, 0.031]).

Taken together, the mediation results indicate that perceived stereotype accessibility plays a critical psychological translating role in the relationship between media exposure and national attitudes. However, this mediating process operates primarily through the competence dimension of national stereotype content rather than the warmth dimension, revealing a clear dimension-specific pattern in the psychological mechanisms through which media exposure shapes national attitudes.

After establishing the moderating role of media literacy in the relationship between media exposure and perceived stereotype accessibility, we further examined whether media literacy moderated the indirect effects through which media exposure influenced national stereotype content and overall attitudes via perceived stereotype accessibility. Given that media literacy was hypothesized to moderate the path from media exposure to perceived stereotype accessibility, conditional indirect effects were estimated using a bootstrapping procedure with 5,000 resamples at different levels of media literacy.

As shown in Table 7, media literacy significantly moderated the indirect effect along the competence stereotype pathway. At lower levels of media literacy (−1 SD), media exposure exerted a significant indirect effect on competence stereotypes through perceived stereotype accessibility. At higher levels of media literacy (+1 SD), this indirect effect remained statistically significant but was substantially weaker in magnitude. A similar pattern emerged when overall national attitude was specified as the outcome variable. The serial indirect effect of media exposure on overall attitude through perceived stereotype accessibility and competence stereotypes was significantly stronger under low media literacy than under high media literacy.

In contrast, no evidence of moderated mediation was observed for the warmth stereotype pathway. Across both low and high levels of media literacy, the indirect effect of media exposure on warmth stereotypes via perceived stereotype accessibility did not reach statistical significance. Consistent with this result, the corresponding serial indirect effects through warmth stereotypes on overall national attitude were also non-significant at all levels of media literacy.

### 4.3 Results of study 3

#### 4.3.1 Objective

Following the experimental evidence obtained in Study 2, Study 3 employed a two-wave short-term panel design to examine whether earlier media exposure predicted later perceived stereotype accessibility and national evaluations under naturally occurring media exposure. The two waves were separated by approximately 2 weeks. Accordingly, the study was intended to examine short-term time-lagged relationships rather than long-term change or enduring temporal stability.

#### 4.3.2 Method

Study 3 employed a two-wave short-term panel design to examine the time-lagged relationships among media exposure, perceived stereotype accessibility, national stereotype content, and national attitudes. Participants were Chinese university students who voluntarily took part in the study through WJX.COM. The platform was used to administer both waves of the survey and record completion times. All participants were informed of the study purpose, anonymity procedures, and their right to withdraw at any time prior to participation.

At Time 1 (T1), a total of 334 questionnaires were collected. After excluding responses with unusually short completion times, failed attention checks, or evident response patterns indicative of low data quality, 317 valid responses were retained. Measures at T1 included Germany-related media exposure, media literacy, competence stereotypes, warmth stereotypes, and overall attitudes toward Germany. These baseline measures were used to account for prior national evaluations before the follow-up assessment. Demographic and background information was also collected, including gender, age, academic year, field of study, German language learning experience, prior visits to Germany, direct contact with German individuals, and attention to international news.

At Time 2 (T2), conducted approximately 2 weeks after T1, participants who completed the first survey were invited to complete a follow-up questionnaire. At Time 2, participants completed the measure of perceived stereotype accessibility and repeated the measures of competence stereotypes, warmth stereotypes, and overall attitudes toward Germany. A total of 259 participants completed both waves, yielding a retention rate of approximately 81.7%. Attrition was primarily due to scheduling conflicts or non-response. Comparisons between retained and attrited participants on key T1 variables revealed no significant differences, suggesting that sample attrition did not introduce systematic bias.

A time-lagged structural model was estimated in which Time 1 media exposure predicted Time 2 perceived stereotype accessibility, which subsequently predicted Time 2 competence, warmth, and overall attitudes. Baseline competence, warmth, and overall attitude measures were included as controls. Before estimating the structural paths, measurement invariance across the two waves was examined for competence, warmth, and overall attitude toward Germany. Configural, metric, scalar, and partial scalar invariance models were evaluated sequentially. Indirect and conditional indirect effects were estimated using 5,000 bootstrap resamples.

#### 4.3.3 Results

Measurement invariance across the two waves was examined sequentially for competence, warmth, and overall attitude toward Germany. As shown in Table 8, the configural and metric invariance models demonstrated acceptable fit, and the changes in CFI and RMSEA supported equivalence of the factor structure and factor loadings across the two waves. The full scalar invariance model produced a somewhat greater deterioration in fit, with ΔCFI slightly exceeding the recommended threshold of 0.010. Inspection of the modification indices indicated that the intercepts of one warmth item and one overall-attitude item differed across waves. After releasing these two intercept constraints, the partial scalar invariance model showed acceptable fit, with ΔCFI = 0.005 and ΔRMSEA = 0.001 relative to the metric model. These results supported partial scalar invariance, indicating that the repeated measures were sufficiently comparable across the two waves for the subsequent structural analyses.

**Table 8:** Longitudinal measurement invariance results.

Model	χ^2^	df	CFI	TLI	RMSEA	SRMR	ΔCFI	ΔRMSEA
Configural invariance	463.18	318	0.949	0.940	0.042	0.046	—	—
Metric invariance	477.92	326	0.947	0.942	0.043	0.051	0.002	0.001
Scalar invariance	526.84	334	0.935	0.932	0.047	0.060	0.012	0.004
Partial scalar invariance	498.76	332	0.942	0.938	0.044	0.055	0.005	0.001

Given the support for configural, metric, and partial scalar invariance, the time-lagged structural model was subsequently estimated. The model showed acceptable fit, χ^2^/df = 2.21, CFI = 0.93, TLI = 0.92, RMSEA = 0.067, and SRMR = 0.061.

As shown in Table 9, Time 1 media exposure significantly and positively predicted Time 2 perceived stereotype accessibility (β = 0.312, SE = 0.058, *p* < 0.001). This result indicates that the association between media exposure and the perceived accessibility of national stereotype-related representations remained evident when the predictor and mediator were measured at separate time points. Time 2 perceived stereotype accessibility, in turn, significantly predicted competence stereotypes (β = 0.421, SE = 0.064, *p* < 0.001), whereas its relationship with warmth stereotypes was weaker and non-significant (β = 0.098, SE = 0.071, *p* = 0.167).

**Table 9:** Time-lagged structural model of Study 3.

Path	β	SE	*p*	Significant
ME → SA	0.312	0.058	<0.001	H1 Significant
SA → CO	0.421	0.064	<0.001	H2a Significant
SA → WA	0.098	0.071	0.167	H2b Not Significant
CO → AT	0.389	0.069	<0.001	H3a Significant
WA → AT	0.164	0.083	0.052	H3b Marginal significance
SA → AT	0.041	0.057	0.471	—
ME → AT	0.028	0.054	0.612	—
ME×ML → SA	−0.142	0.056	0.011	H5 Significant

Competence stereotypes significantly predicted overall attitudes toward Germany (β = 0.389, SE = 0.069, *p* < 0.001). The effect of warmth stereotypes was weaker and marginally significant (β = 0.164, SE = 0.083, *p* = 0.052). Neither perceived stereotype accessibility nor media exposure had a significant direct effect on overall attitudes after stereotype content was included in the model. Media literacy significantly moderated the relationship between media exposure and perceived stereotype accessibility (β = −0.142, SE = 0.056, *p* = 0.011), indicating that this association was weaker among participants with higher media literacy.

Indirect effects were subsequently estimated using 5,000 bootstrap resamples. As reported in Table 10, media exposure had a significant indirect effect on competence stereotypes through perceived stereotype accessibility (indirect effect = 0.131, SE = 0.039, 95% CI [0.067, 0.212]). The corresponding indirect effect on warmth stereotypes was not significant because the confidence interval included zero.

**Table 10:** Indirect effects and moderating mediating effects of Study 3 (Bootstrap = 5,000).

Indirect path	Indirect effect	SE	95% CI	Significant
ME → SA → CO	0.131	0.039	[0.067, 0.212]	H4a Significant
ME → SA → WA	0.031	0.028	[−0.018, 0.092]	H4b Not Significant
ME → SA → CO → AT	0.083	0.027	[0.039, 0.148]	—
ME → SA → WA → AT	0.012	0.015	[−0.014, 0.046]	—
ME → SA → AT	0.006	0.012	[−0.018, 0.031]	—

Indirect path(T1 → T2)	Media literacy level	Conditional indirect effect	SE	95% CI	Significant
ME (T1) → SA (T2) → CO (T2)	Low ML (−1 SD)	0.154	0.041	[0.081, 0.241]	Significant
	High ML (+1 SD)	0.091	0.036	[0.028, 0.165]	Significant
ME (T1) → SA (T2) → WA (T2)	Low ML (−1 SD)	0.047	0.033	[−0.009, 0.118]	Not Significant
	High ML (+1 SD)	0.019	0.026	[−0.021, 0.082]	Not Significant
ME (T1) → SA (T2) → CO (T2) → AT (T2)	Low ML (−1 SD)	0.100	0.031	[0.048, 0.173]	Significant
	High ML (+1 SD)	0.058	0.024	[0.018, 0.116]	Significant
ME (T1) → SA (T2) → WA (T2) → AT (T2)	Low ML (−1 SD)	0.016	0.016	[−0.010, 0.052]	Not Significant
	High ML (+1 SD)	0.007	0.011	[−0.015, 0.034]	Not Significant

Indirect effects were evaluated using 5,000 bootstrap resamples; effects were considered statistically significant when the 95% confidence interval excluded zero.

The serial indirect pathway from media exposure to overall attitudes through perceived stereotype accessibility and competence stereotypes was significant (indirect effect = 0.083, SE = 0.027, 95% CI [0.039, 0.148]). In contrast, the serial indirect pathway through warmth stereotypes and the single-mediator pathway through perceived stereotype accessibility alone were not significant. These findings replicated the dimension-specific pattern observed in Study 2 under short-term temporal separation.

## 5 Conclusion

### 5.1 Discussion and theoretical implications

In highly mediatized environments, individuals increasingly form perceptions of foreign countries through indirect media exposure rather than direct interaction. Although communication studies and political psychology have extensively examined how media narratives shape national images (Kertzer and Zeitzoff, 2017; Lăpădat, 2022), existing research has focused primarily on media framing and aggregate opinion outcomes. Less attention has been paid to the individual-level cognitive process through which media information is translated into national stereotype content and subsequent attitudes. By combining measurement development, experimental testing, and short-term panel evidence, the present research provides a social-cognitive account of this process.

Across the experimental and short-term panel studies, media exposure showed no significant direct effect on overall attitudes toward Germany. Instead, its influence operated through perceived stereotype accessibility and subsequent stereotype content, suggesting that media shape which nation-related representations are readily retrieved during judgment rather than directly prescribing positive or negative evaluations (Higgins and Eitam, 2014; González-Bailón and Lelkes, 2023).

The first theoretical contribution lies in identifying perceived stereotype accessibility as a distinct cognitive link between media exposure and national evaluation. Previous research on stereotype accessibility has concentrated mainly on relatively concrete categories, such as ethnic, gender, and occupational groups (Gawronski and Bodenhausen, 2015). Nations, however, are more abstract collective targets and are often encountered without sustained direct contact. The present findings show that repeated media exposure can increase the perceived accessibility of nation-related representations, which subsequently provide cognitive inputs for national evaluation. Perceived stereotype accessibility reflects the subjective ease with which stereotype-related impressions come to mind, whereas competence and warmth capture the substantive content of those impressions. This account complements cultivation, agenda-setting, and framing theories, which explain how repeated exposure, issue salience, and attribute emphasis structure the information audiences encounter (Entman, 2007; Flusberg et al., 2024; McCombs, 2005; Morgan et al., 2015). The present study extends these perspectives by identifying the individual-level cognitive process through which such information becomes available for subsequent national evaluation.

Second, the findings refine the stereotype content model by revealing a consistent dimension-specific process. Perceived stereotype accessibility predicted competence stereotypes in both Study 2 and Study 3, whereas its relationship with warmth stereotypes was not significant. Germany-related media frequently emphasize economic performance, technological capability, manufacturing, education, and institutional effectiveness, which provide relatively direct cues for competence judgments. Warmth, by contrast, concerns friendliness, intentions, emotional closeness, and interpersonal approachability and may depend more strongly on relational and affective cues emphasized in parasocial contact and mediated intergroup contact research (Joyce and Harwood, 2014; Ortiz and Harwood, 2007; Schiappa et al., 2005). For Chinese university students with limited direct experience of Germany, competence may therefore provide a more available basis for national evaluation, while the weak warmth pathway may reflect the limited contact-like information contained in general media exposure. Social identity processes may also shape these evaluations through comparisons between national ingroups and outgroups (Hornsey, 2008). The observed asymmetry may thus reflect the combined influence of Germany’s competence-oriented media image, the information encountered by participants, and their limited direct contact with the country.

Third, the study extends the theoretical role of media literacy by identifying the stage at which it constrains media influence. Media literacy did not directly determine stereotype content or overall attitudes. Instead, it weakened the relationship between media exposure and perceived stereotype accessibility. This pattern suggests that media literacy functions as an early-stage cognitive filter that reduces the likelihood that repeatedly encountered or prominently framed media representations will become dominant cues in subsequent national judgments. The findings therefore integrate media literacy into a social-cognitive model of stereotype formation rather than treating it solely as a general communication competence or intervention outcome.

### 5.2 Practical implications

The findings offer several practical implications for national image communication, media production, and media literacy education. First, organizations involved in public diplomacy and international communication should recognize that increasing the volume of media exposure may not directly improve national attitudes. What matters is the type of national representation that becomes cognitively accessible. Repeated exposure to information about Germany’s industrial capacity, technological development, vocational education, and institutional efficiency may strengthen competence-related images, but it is less likely to improve perceptions of warmth. National image strategies should therefore move beyond increasing general visibility and consider which specific attributes are repeatedly emphasized across media content.

Second, the asymmetric effects of competence and warmth suggest that Germany-related communication may benefit from a more balanced representational strategy. Existing media narratives often portray Germany as efficient, technologically advanced, disciplined, and institutionally capable. Although these attributes support a strong competence image, they may provide limited information about friendliness, emotional accessibility, and everyday social life. Communication aimed at strengthening warmth perceptions could place greater emphasis on interpersonal narratives, ordinary citizens, cultural participation, hospitality, community life, and collaborative experiences between Chinese and German individuals. Such content may provide the relational and affective cues needed for warmth judgments rather than merely reinforcing an already established competence image.

Third, the findings have implications for media organizations and content producers. News reports and digital platforms do not simply communicate factual information; through repetition and attribute emphasis, they also influence which national representations become readily available during judgment. Media practitioners should therefore be aware that persistent concentration on a narrow set of attributes may produce simplified or one-dimensional national images. A broader range of economic, social, cultural, and interpersonal content could help audiences form more differentiated perceptions of foreign countries.

Finally, the moderating role of media literacy suggests that media education should address not only misinformation or source credibility but also the cognitive consequences of repeated exposure. Individuals may rely on frequently encountered national representations even when such information is not explicitly persuasive. Media literacy programs could therefore help audiences recognize how repetition, selective emphasis, and limited representational diversity shape the information that comes most readily to mind. Encouraging comparison across sources and reflection on absent or underrepresented attributes may reduce dependence on highly accessible media stereotypes and support more autonomous national judgments.

### 5.3 Limitations and future research directions

Several limitations should be acknowledged. First, perceived stereotype accessibility was assessed as a subjective judgment of retrieval ease and judgmental fluency. This self-report approach reflects participants’ perceived ease of bringing stereotype-related impressions to mind. Behavioral measures, such as reaction-time tasks, lexical decision procedures, and sequential priming paradigms, provide a complementary assessment of accessibility based on response performance. Future research could combine these approaches to provide a more comprehensive assessment of stereotype accessibility.

Second, several focal variables were collected through self-report questionnaires, creating a potential risk of common method bias. The experimental manipulation in Study 2 and the temporal separation introduced in Study 3 reduce, but do not eliminate, this concern. Future studies could combine survey responses with behavioral measures, media-use records, content-tracking data, or assessments collected from different sources and at multiple time points.

Third, the studies focused on Chinese university students’ perceptions of Germany. The relatively homogeneous age and educational background of the participants may limit the generalizability of the findings, while their limited direct contact with Germany may have increased their reliance on mediated information. Moreover, the current design cannot determine whether the stronger competence pathway reflects Germany’s specific media image, characteristics of Chinese perceivers, or an interaction between the target country and the cultural context. Comparative studies involving more diverse populations, different cultural audiences, and multiple national targets are needed to clarify the scope of the competence–warmth asymmetry.

Fourth, the experimental materials in Study 2 were researcher-developed and presented under controlled conditions. Although the texts were matched in length, format, linguistic complexity, and evaluative tone, they cannot fully reproduce the multimodal, selective, and interactive nature of everyday media consumption. Participants may also have processed the materials differently from naturally encountered news and social media content. Future research could use authentic media materials, field experiments, platform-based exposure data, or designs that distinguish among news, entertainment, and social media content. It could also directly compare stereotype-consistent and counter-stereotypical information to examine how different types of content shape the relationship between media exposure and perceived stereotype accessibility.

Finally, Study 3 employed a two-wave short-term panel design with an interval of approximately 2 weeks. This design introduced temporal separation between the proposed antecedents and subsequent outcomes but did not capture long-term change, cumulative media influence, or reciprocal relationships among the constructs. Longer intervals and three or more measurement waves would allow future research to examine within-person change and potential reciprocal relationships among media exposure, perceived stereotype accessibility, stereotype content, and national attitudes.

## Data Availability

The raw data supporting the conclusions of this article will be made available by the authors, without undue reservation.
