# Supplementary material for: Media exposure, perceived stereotype accessibility, and national image formation: experimental and short-term panel evidence from Chinese perceptions of Germany
Source: Front Psychol. 2026 Sep 4;17:1858109. doi: 10.3389/fpsyg.2026.1858109 (PMC13586008; doi:10.3389/fpsyg.2026.1858109)
Supplement: Supplementary file 1 [file Supplementary_file_1.docx]

**Appendix A**

**(1) Media Exposure to Germany**

Items (4 items)

ME1. I frequently encounter content related to Germany or Germans in the media.

ME2. Media serve as an important source of information for my understanding of Germany.

ME3. I can easily recall media content about Germany that I have seen recently.

ME4. Information related to Germany occupies a noticeable proportion of my overall media exposure.

**(2) Perceived stereotype accessibility**

Items (4 items)

SA1. When I think of Germans, certain typical characteristics immediately come to mind.

SA2. My impression of Germans is clear and well defined.

SA3. It is not difficult for me to describe what a typical German is like.

SA4. When I need to evaluate Germans, I can form a judgment very quickly.

**(3) National Stereotype Content: Competence**

Items (4 items)

CO1. I believe that Germans are generally efficient in their work and in handling affairs.

CO2. German society gives an impression of professionalism and competence.

CO3. Germans demonstrate strong abilities in fields such as technology or engineering.

CO4. Overall, I consider Germany to be a country that operates efficiently.

**(4) National Stereotype Content: Warmth**

Items (4 items)

WA1. I believe that Germans are generally friendly.

WA2. German society as a whole gives a sense of trustworthiness.

WA3. Interacting with Germans makes me feel comfortable.

WA4. Germans usually appear cold or distant. (reverse coded)

**(5) Overall Attitude Toward Germany**

Items (3 items)

AT1. Overall, I have developed a relatively positive impression of Germany.

AT2. I hold a favorable attitude toward Germany as a country.

AT3. I believe that Germany is a country worthy of respect.

**(6) Media Literacy**

Items (5 items)

ML1. I usually reflect on the positions and intentions behind media messages.

ML2. I am aware that media content does not necessarily fully reflect reality.

ML3. I compare information from different sources rather than relying on a single account.

ML4. I question the way media portray countries or social groups.

ML5. Even when the media repeatedly present a certain image, I maintain my own judgment.

**Appendix B**

**Sample Experimental Materials for Study 2**

The experimental materials were originally presented in Chinese. The English versions below are provided for reporting purposes. The passages were developed by the researchers through the synthesis and paraphrasing of publicly available factual reports and news materials. They were written in a neutral and descriptive style, and source names, logos, and identifying information were removed.

**Sample Germany-related paragraph**

Germany’s economy includes a large manufacturing sector alongside service industries. Automobile production, mechanical engineering, chemicals, and business services contribute to employment and international trade. Public discussion has also addressed energy costs, demographic change, and the adjustment of industrial production. These developments have created both opportunities and pressures for German firms and public institutions.

**Sample neutral filler paragraph**

Urban public transportation connects residential areas, workplaces, schools, and commercial districts. Common systems include buses, underground trains, suburban railways, and shared bicycles. Transportation planning involves decisions about routes, passenger capacity, frequency, ticket prices, and accessibility. Digital applications increasingly provide passengers with information about arrival times and service changes.
